# Surface Energy‐Assisted Patterning of Vapor Deposited All‐Inorganic Perovskite Arrays for Wearable Optoelectronics

**DOI:** 10.1002/advs.202402635

**Published:** 2024-04-19

**Authors:** Zhangsheng Xu, Xiaojun Pan, Hui Lu, Qiuchun Lu, Yegang Liang, Zeping He, Yizhi Zhu, Yang Yu, Wenqiang Wu, Xun Han, Caofeng Pan

**Affiliations:** ^1^ Beijing Institute of Nanoenergy and Nanosystems Chinese Academy of Sciences Beijing 101400 P. R. China; ^2^ School of Nanoscience and Engineering University of Chinese Academy of Sciences Beijing 100049 P. R. China; ^3^ Institute of Microscale Optoelectronics Shenzhen University Shenzhen 518060 P. R. China; ^4^ Department of Applied Physics The Hong Kong Polytechnic University Hong Kong 999077 P. R. China; ^5^ Institute of Atomic Manufacturing Beihang University Beijing 100191 P. R. China

**Keywords:** all‐inorganic perovskite, photodetector arrays, pulse sensing, surface energy, vapor deposition

## Abstract

Solution‐based methods for fabricating all‐inorganic perovskite film arrays often suffer from limited control over nucleation and crystallization, resulting in poor homogeneity and coverage. To improve film quality, advanced vapor deposition techniques are employed for continuous film. Here, the vapor deposition strategy to the all‐inorganic perovskite films array, enabling area‐selective deposition of perovskite through substrate modulation is expanded. It can yield a high‐quality perovskite film array with different pixel shapes, various perovskite compositions, and a high resolution of 423 dpi. The resulting photodetector arrays exhibit remarkable optoelectronic performance with an on/off ratio of 13 887 and responsivity of 47.5 A W^−1^. The device also displays long‐term stability in a damp condition for up to 12 h. Moreover, a pulse monitoring sensor based on the perovskite films array demonstrates stable monitoring for pulse signals after being worn for 12 h and with a low illumination of 0.055 mW cm^−2^, highlighting the potential application in wearable optoelectronic devices.

## Graphical abstract

Through the surface energy regulation strategy, high‐quality and high‐resolution all‐inorganic perovskite film arrays are successfully manufactured. This approach enabled the preparation of a high‐performance perovskite photodetector array with excellent photoelectric performance and stability. It can be integrated into pulse monitoring systems and is capable of stably detecting pulse signals even after continuous wearing and under low light intensity.

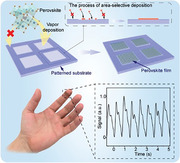

## 1 Introduction

Metal halide perovskite has shown great potential in the field of optoelectronic devices, including photodetectors,^[^ ^1^, ^2^, ^3^, ^4^, ^5^, ^6^, ^7^, ^8^, ^9^ ^]^ light‐emitting diodes,^[^ ^10^, ^11^, ^12^, ^13^ ^]^ and solar cells,^[^ ^14^, ^15^, ^16^, ^17^, ^18^, ^19^, ^20^, ^21^, ^22^ ^]^ owing to its long carrier lifetime, diffusion length, tunable bandgap, and high absorption coefficient.^[^ ^23^, ^24^, ^25^, ^26^ ^]^ Precisely controlled growth of perovskite arrays is of great significance in the preparation and integration of high‐performance optoelectronic devices.^[^ ^27^ ^]^ Due to poor stability and solubility in common solvents, the traditional lithography process is not suitable for perovskite materials.^[^ ^28^ ^]^ In recent years, various methods for controlled synthesis of perovskite arrays have been proposed, such as the one‐ or two‐step spin‐coating method,^[^ ^3^, ^29^ ^]^ inkjet printing method,^[^ ^30^, ^31^, ^32^ ^]^ and template‐assisted patterning.^[^ ^1^, ^33^ ^]^ However, most of the preparation methods are solution‐based and only applicable to organic‐inorganic hybrid perovskite materials, which suffer from poor environmental stability.^[^ ^34^, ^35^, ^36^ ^]^ In contrast, all‐inorganic perovskite achieves higher stability by replacing organic cations (MA^+^/FA^+^) with inorganic cations (Cs^+^), while maintaining excellent photoelectric performance.^[^ ^37^, ^38^, ^39^ ^]^

Several fabrication strategies of all‐inorganic perovskite arrays have been reported. For example, photolithographic templates or shadow mask‐assisted methods combined with the vapor deposition process are utilized to generate different CsPbBr_3_ patterns.^[^ ^40^, ^41^ ^]^ However, these methods could suffer from pattern distortion when the feature size of individual pixels is reduced to below hundreds of microns, due to the shadow effect from the mask height and pixel damage in the template lift‐off process. Recently, the mask‐free surface modification method and direct inkjet printing have been proposed for CsPbBr_3_ polycrystalline film and single crystal arrays, promising for easy integration of large‐scale devices.^[^ ^31^, ^42^, ^43^ ^]^ Due to the low solubility of all‐inorganic perovskite and the low volatility of solvent, these solution‐based methods usually demonstrated arrays with small fill factors. Compared to the solution‐based method,^[^ ^44^ ^]^ the vapor deposition method, as a solvent‐free method, has been demonstrated to be an effective technique for controlling the morphology of perovskite film, reducing surface defects, improving crystallization quality, and preventing solvent residue.^[^ ^45^, ^46^, ^47^ ^]^ Therefore, combining the advantages of mask‐free strategy and vapor deposition is critical for achieving high‐fidelity and high‐resolution all‐inorganic perovskite arrays. Currently, it is challenging to regulate the nucleation and crystallization process of the all‐inorganic perovskite arrays in the vapor deposition process.

In this work, we focus on the influence of surface energy on the nucleation and growth during vapor deposition processes and propose a surface energy‐assisted patterning and vapor deposition (SEAPVD) strategy for the fabrication of the all‐inorganic perovskite films array. Through modulating surface energy on the different areas by surface treatment, all‐inorganic perovskite films can be deposited on the designed patterns, resolutions, and dimensions. The perovskite film array exhibits excellent stability after storing in the air for one month. Moreover, the photodetectors array based on perovskite prepared by our method exhibit outstanding photoelectric performance with a high on/off ratio of 13 877, fast response (0.81 /2.03 ms), long‐term tolerance in the high‐humidity environment for 12 h. It could serve as a sensing element in a flexible pulse monitoring system, enabling stable pulse monitoring at rest or after exercise and after being continuously worn for 12 h, and the pulse signal can be monitored with a weak light source intensity of 0.055 mW cm^−2^. This approach offers precise control of the high‐quality all‐inorganic perovskite film array, which has potential applications in integrated wearable optoelectronics.

## 2 Results and Discussion

### 2.1 Fabrication and Characterization of All‐Inorganic Perovskite Arrays

**Figure 1:**
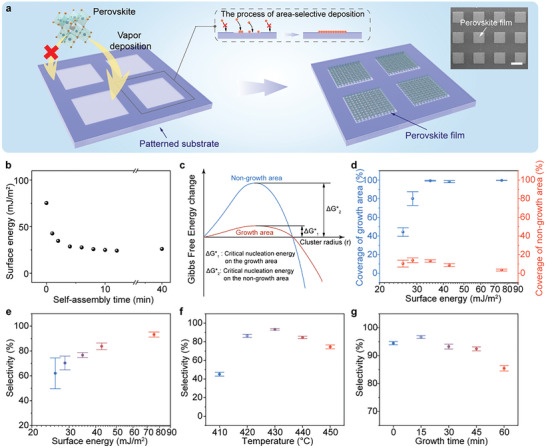
Vapor deposition of perovskite films array. a) Schematic diagram of vapor deposition of perovskite films array. The inset is SEM images of the as‐fabricated perovskite films array. The scale bar is 100 µm. b) The plot of surface energy versus self‐assembly time. c) Schematic illustration of Gibbs free energy change and critical nucleation energy during the deposition process on the growth area and the non‐growth area. d) The dependence of coverage on the growth area (blue) and the non‐growth area (red) on surface energy. e) The dependence of selectivity on surface energy. The dependence of selectivity on f) growth temperature and g) growth time.

**Figure** **1**a schematically illustrates the area‐selective deposition process of the all‐inorganic perovskite films array modulated by the surface energy. The perovskite powder was placed in the center of a tubular furnace as the evaporation source while the glass substrate or polyimide (PI) film served as the deposition target substrate downstream (Figure S1, Supporting Information). Before deposition, the target substrate was treated through a surface functionalization process to generate variation in surface energy. Through regulating the self‐assembly time of octadecyl‐trichlorosilane (OTS), the surface energy of the target substrate could be modulated in a wide range from 25.8 to 75.4 mJ m^−2^ (Figure 1b), which can be calculated through contact angles and the surface free energy of the known liquids (Note S1 and Table S1, Supporting Information). Details about the substrate treatment process and the regulation of its surface energy can be found in Note S2 and Figure S2–S5 (Supporting Information). With increasing the temperature of the furnace, the perovskite powder sublimated and was carried to the target substrate by argon stream, followed by the nucleation and crystal growth of the perovskite. The high surface energy of the target substrate could promote the heterogeneous nucleation of perovskite due to that it reduces the Gibbs free energy for nucleation (Figure 1c).^[^ ^48^ ^]^ Thus, by controlling the surface energy to facilitate the heterogeneous nucleation, the perovskite could be deposited at desired locations with various pixel dimensions and resolutions, resulting in the area‐selective deposition of perovskite film.

To investigate the effect of different conditions on the perovskite films array, we defined the coverage (N) and selectivity (S). The coverage is the area ratio of perovskite to the corresponding growth or non‐growth region. The growth region refers to the place treated with oxygen plasma or OTS for perovskite deposition, while the non‐growth region is the gap between growth regions with relatively low surface energy. The selectivity can be calculated by the equation^[^ ^46^, ^49^ ^,^ ^50^ ^]^:

(1) $$S=\frac{N_{growth}-N_{non-gorwth}}{N_{growth}+N_{non-gorwth}}$$

where *N_growth_* and *N_non‐growth_* are the coverage of the growth and non‐growth regions, respectively.

The deposition process of the perovskite films array is controlled by three key parameters, including surface energy, growth temperature, and growth time. Surface energy determines the properties of the perovskite array, such as resolution, pixel dimensions, and shapes, while the growth temperature and time together regulate the morphology of the perovskite film, including grain size, film thickness, and pinholes. Figure 1d demonstrates the coverage of the growth (blue) and non‐growth (red) regions versus the surface energy. With rising surface energy in the growth region, the coverage of the growth area increases rapidly from 44.2% to 99.7%, which is caused by the reduction of the critical nucleation energy barrier. However, coverage of the non‐growth area shows a decreasing trend. It could be observed from the scanning electron microscope (SEM) images of an individual pixel (Figure S6, Supporting Information). The selectivity of the perovskite array was then calculated, as shown in Figure 1e. As the surface energy increased from 25.8 to 75.4 mJ m^−2^, the selectivity was enhanced from 62.1% to 93.3%, realizing the control of the deposition position of the perovskite film array. Figure S7 (Supporting Information) illustrates the dependence of the coverage on the growth temperature. With the increase in the growth temperature, the coverage of the growth region improves rapidly and saturates to nearly 100%. However, the coverage of the non‐growth region shows a reduction at low temperatures and an increase at high temperatures, indicating a lack of nucleation control by the surface energy at high‐temperature conditions. As a result, the maximum value of selectivity of the perovskite film array is obtained at 430 °C (Figure 1f). The growth time demonstrates the different trends in film coverage. As shown in Figure S8 (Supporting Information), the coverage in either the growth or non‐growth region exhibits a monotonic increase with the growth time, which is associated with the crystal growth process. The selectivity demonstrates a maximum value of 96.7% at the growth time of 15 min (Figure 1g). However, some pinholes exist in the 15‐min growth perovskite film (Figure S9a, Supporting Information). In contrast, the 30‐min growth samples show a compact and pinholes‐free surface with a coverage of 99.67%; meanwhile, the coverage of the non‐growth region is below 5% (Figure S9b,c, Supporting Information). Moreover, the extension of growth time leads to an increase of grain size from 1.02 to 2.84 µm (Figure S10, Supporting Information), and the thickness of the perovskite film increased from 0.94 to 2.99 µm (Figure S11, Supporting Information). Both of them exhibit a linear relationship with the growth time, indicating the rapid process of grain consolidation and growth. Compromising the high selectivity and pinhole‐free morphology of the perovskite film array, samples synthesized at 430 °C for 30 min are considered for subsequent device fabrication.

**Figure 2:**
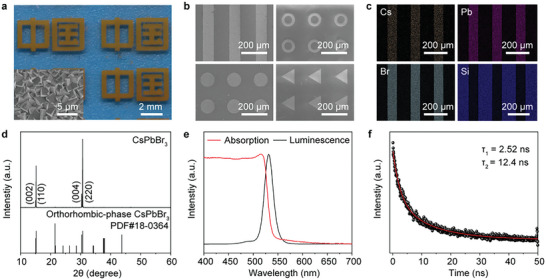
Characterization of the patterned perovskite films. a) Photograph of the perovskite patterns of Chinese characters. The inset is the SEM image of the perovskite film. b) SEM images of perovskite films with the pattern of stripe, ring, circle, and triangle. c) EDS mapping of the perovskite stripes. d) X‐ray diffraction spectrum of CsPbBr_3_ film and its corresponding PDF card. e) PL and absorption spectrum of CsPbBr_3_ film. f) TRPL spectrum of CsPbBr_3_ film.

Based on the SEAPVD method, perovskite films were synthesized with different patterns. As shown in **Figure** **2**a, the perovskite film with the pattern of Chinese characters was deposited. The inset displays the morphology of a perovskite film composed of micron‐sized grains. The total thickness of the film is about 2 µm (Figure S12, Supporting Information). Various patterns (long stripes, rings, circles, triangles) with a pitch of 200 µm were successfully fabricated as shown in Figure 2b. High‐density perovskite film array was also achieved with a resolution of 423 dpi (Figure S13, Supporting Information), demonstrating the potential application of the SEAPVD method in high‐resolution device integration. The chemical composition and structure of the perovskite films array were characterized by energy‐dispersive X‐ray spectroscopy (EDS) and X‐ray diffraction (XRD), respectively. Figure 2c exhibits the EDS mapping results of the perovskite stripes. The Si signal comes from the SiO_2_ pattern in the surface functionalization process. It was observed that the Cs, Pb, and Br elements are uniformly distributed in the entire film and there is no overlap between the perovskite elements and Si, indicating excellent selectivity of the perovskite patterns. Besides, the atomic ratio of Cs: Pb: Br is 1.14: 1: 3.02, close to the ideal element ratio of CsPbBr_3_ (Figure S14, Supporting Information). Figure 2d shows XRD patterns of the as‐fabricated CsPbBr_3_ arrays with peaks in 15°, 15.2°, 30.4°, and 30.7° that correspond to (002), (110), (004), and (220) planes of orthorhombic CsPbBr_3_ (PDF#18‐0364).^[^ ^51^ ^]^ The optical properties of the perovskite array were obtained by photoluminescence (PL) spectroscopy, absorption spectrum, and time‐resolved photoluminescence (TRPL) spectroscopy. As shown in Figure 2e, the PL peak and the edge absorption of the as‐fabricated CsPbBr_3_ film were observed at 531 and 537 nm, respectively, which is consistent with the previously reported CsPbBr_3_.^[^ ^52^, ^53^ ^]^ The PL lifetime of the CsPbBr_3_ film could be extracted by fitting the PL decaying curve with the exponential function. As shown in Figure 2f, the lifetime exhibits a fast component of 2.52 ns and a slow component of 12.4 ns, which can be considered as surface and bulk recombination,^[^ ^54^ ^]^ respectively. Moreover, by regulating the proportion of halogen elements in the evaporation source, CsPbX_3_ film arrays with different halogen ratios could be fabricated (Figure S15a, Supporting Information). Figure S15b (Supporting Information) exhibits the SEM images of CsPbCl_3_, CsPbCl_2_Br, CsPbClBr_2_, CsPbBr_3_, CsPbBr_2_I, CsPbBrI_2_, CsPbI_3_ arrays, respectively. The corresponding micrographs are displayed in Figure S15c (Supporting Information). As shown in Figure S15d (Supporting Information), XRD analysis reveals that an increase in Cl elements or a decrease in I elements led to a red shift of the peaks ascribed to (002)/(110) and (004)/(220). Figure S15e (Supporting Information) exhibits the PL peaks of CsPbX_3_ centered at 416, 446, 488, 531, 570, 640, and 712 nm. The absorption spectrum in Figure S15f (Supporting Information) demonstrates that the bandgap of CsPbX_3_ could be modulated from 2.98 to 2.31 eV with the substitution of Cl by Br and from 2.31 to 1.74 eV with the substitution of Br by I. These results reveal the modulation ability of the SEAPVD method including deposition position, grain size, resolution, and composition of perovskite, demonstrating the potential application in integrated optoelectronics.

### 2.2 Optoelectronic Performance of The Perovskite Photodetector

**Figure 3:**
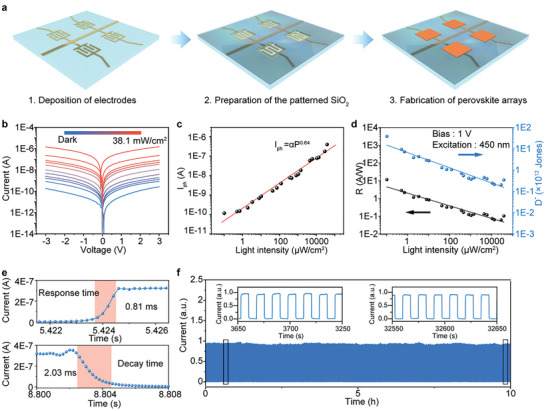
Performance characterization of the perovskite photodetector prepared through the SEAPVD method. a) Schematic diagram of the fabrication process of the perovskite photodetector arrays. b) I‐V curves of perovskite photodetector under 450 nm illumination with light intensities varying from 0 to 38.1 mW cm^−2^. c) Dependence of *I_ph_* on light intensity. d) Dependence of responsivity and detectivity on light intensity. e) Response time and decay time of the perovskite photodetector. f) I‐T curves of the device for 10 h under 450 nm illumination.

Due to the capability to control the position and dimensions of the perovskite films array, the SEAPVD method provided an easy and solvent‐free strategy for the integration of high‐performance optoelectronic device arrays. A flexible photodetectors array was demonstrated on a PI substrate with the photoconductor structure. **Figure** **3**a schematically illustrates the fabrication process of the array. It started with the Au interdigital electrodes deposition on the substrate and then the SiO_2_ patterns aligned with Au electrodes were prepared, followed by the surface functionalization. Subsequently, perovskite film arrays were synthesized on the Au electrodes through the SEAPVD method. The perovskite film of the device demonstrates a textured structure (Figure S16, Supporting Information). The light could be reflected between the facets of the textured surface and bound within perovskite film, which reduces reflection loss and promotes light absorption.^[^ ^55^ ^]^ Figure 3b presents the typical current‐voltage (I‐V) curves of the photodetectors array under 450 nm illumination with intensities ranging from 0 to 38.1 mW cm^−2^. With the increase of light intensity, the current of the device at 1 V bias is significantly enhanced from 29 pA (under the dark condition) to 397 nA (38.1 mW cm^−2^), indicating a high on/off ratio of 13 887. Figure 3c demonstrates the dependence of photocurrent (*I_ph_*) on the light intensity, which can be fitted by the equation^[^ ^56^, ^57^ ^]^:

(2) $$I_{ph}=\alpha P^{\beta }$$

where *P* is light intensity, and *I_ph_* is the difference of current under light and dark conditions, expressed by *I_ph_ = I_light_ – I_dark_*. *β* is calculated to be 0.64, showing the sublinear relationship between *I_ph_* and light intensity. Linear dynamic range (LDR) can be calculated by equation:^[^ ^58^ ^]^

(3) $$LDR=20\log(\frac{I_{max}}{I_{dark}})$$

where *I_dark_* is the current in the dark and *I_max_* is the maximum photocurrent under the illumination of a specific wavelength, corresponding to the minimum current of 29 pA in the dark and the photocurrent of 397 nA at 38.1 mW cm^−2^. LDR was calculated to be 83 dB. Responsibility (*R*) and detectivity (*D*) are key parameters of the photodetector. They can be calculated according to equation^[^ ^42^ ^]^:

(4) $$R=\frac{I_{ph}}{PA}$$

(5) $$D=\frac{R}{\sqrt{2eJ_{dark}}}$$

where *A*, *e*, and *J_dark_* are the efficient illumination area, the elementary charge, and the current density in the dark, respectively. The dependence of *R* and *D* on light intensity is displayed in Figure 3d. Both *R* and *D* decrease as the light intensity increases, which can be explained by the saturation of light absorption of perovskite when the light intensity is large. Under the illumination of 0.101 µW cm^−2^, *R* and *D* reached maximum values of 11.56 A W^−1^ and 3.83 × 10^13^ Jones at 1 V bias, 47.5 A W^−1^ and 6.24 × 10^13^ Jones at 3 V bias. The response time and decay time refer to the time difference to reach 10% and 90% of the maximum current. As shown in Figure 3e, a response time of 0.81 ms and a decay time of 2.03 ms are obtained. Besides, the perovskite photodetectors array demonstrates excellent working stability, showing unchanged dynamic photo‐switching characteristics after continuous operation for 10 h under the illumination of 1.34 mW cm^−2^. We compared the performance of our device with other previously reported all‐inorganic perovskite photodetectors with the photoconductor structure in Table S2 (Supporting Information), and it is comparable to or even higher than the state‐of‐art devices referring to the responsivity, on/off ratio, and response/decay time.

### 2.3 Stability of the Perovskite Film and the Flexible Photodetector

**Figure 4:**
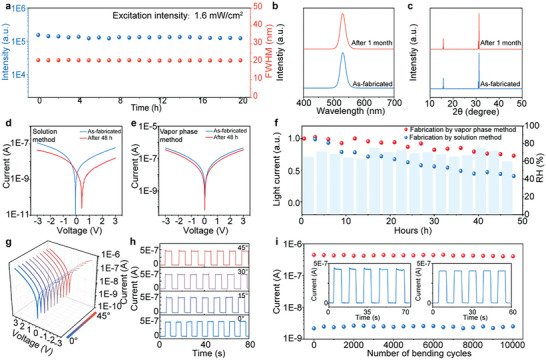
Stability of the perovskite film and photodetector. a) PL intensity and FWHM of perovskite film for 20 h under the illumination of 1.6 mW cm^−2^. b) PL spectrum and c) XRD spectrum of perovskite film as prepared and after a month. I‐V curves of the perovskite photodetector prepared by d) solution method and e) vapor phase method before and after 48 h in a high humidity environment. f) Normalized light current of the device stored in a high humidity environment for 48 h. g) I‐V curves and h) I‐T curves of the device during bending at different angles. i) Dependence of light and dark current on the number of bending cycles. The inset is the I‐T curves of the device before and after 10 000 bending cycles.

The stability of the perovskite could be remarkably improved by the SEAPVD method, attributed to the high crystal quality of the vapor‐deposited perovskite films array. The structural and mechanical stability were carefully characterized, as shown in **Figure** **4**. All the samples are measured in the air without encapsulation. We first investigated the light stability of perovskite films array with 380 nm photoexcitation of 1.6 mW cm^−2^ for 20 h. Figure 4a depicts the PL intensity and full width at half‐maximum (FWHM) of the perovskite films array. Both the PL intensity and FWHM remain unchanged, proving that there is no light‐induced degradation of the perovskite films array. In addition, there was no change in peaks even after storage in the air for 1 month (Figure 4b). Figure 4c compares XRD peaks of the samples as fabricated and stored after 1 month, without observing new peaks and changing of peak positions. These results confirm the excellent structural stability of perovskite films array in the air. Furthermore, the perovskite photodetectors array without encapsulation was exposed to a harsh environment with an average humidity of 68.6%. As a comparison, a device based on the solution‐processed CsPbBr_3_ films array was prepared and measured together. Figure 4d,e depicts I‐V curves of the perovskite photodetectors fabricated with the solution method and vapor phase method under the 450 nm illumination, respectively. After 48 h, the device prepared by the solution method exhibited a noticeable decrease in the light current. Figure 4f exhibits normalized photocurrent depending on storage time, where red dots represent the photocurrent of vapor phase method‐based photodetector; blue dots are from solution method‐based device; and the light blue column is humidity. The vapor phase method‐based device remains a stable photocurrent, while the other device demonstrates a 25% drop of the photocurrent in the first 12 h. Finally, the vapor phase‐based device could maintain over 73% of the initial photocurrent, whereas the other device has dropped to 41%, demonstrating the excellent environmental tolerance of the perovskite photodetector manufactured through the SEAPVD methods.

In addition, the mechanical stability of the device was investigated through the bending test. The flexible device was fixed on a large polyethylene glycol terephthalate (PET) substrate, to which the ends were attached with two moveable stages (Figure S17a, Supporting Information). By tuning the distance between the two stages, the device could be bent at angles ranging from 0° to 45° (Figure S17b, Supporting Information). As shown in Figure 4g, there is no change in the I‐V curve of the photodetectors array under the bending angle of 45°. Additionally, as shown in Figure 4h, the on/off switching properties of the perovskite photodetectors array remain stable under the bending angle of 0–45°, indicating the feasibility of the device to be operated under the large mechanical deformation condition. To further investigate the electronic stability of the photodetectors array during mechanical deformation, the fatigue measurement of the device with a bending angle of 30° was performed. Figure 4i exhibits light currents, dark currents, and light‐switching characteristics after different bending cycles, which remained stable after 10 000 bending cycles, proving the high deformation tolerance of the flexible perovskite photodetectors array.

### 2.4 Pulse Monitoring System Integration and Tests

**Figure 5:**
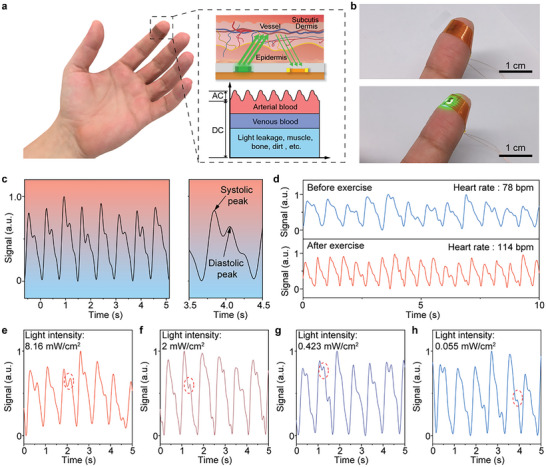
Pulse monitoring system based on a flexible perovskite photodetector array. a) Schematic illustration of the perovskite photodetector‐based PPG sensor. The inset is the working principle diagram of the PPG sensor and the schematic diagram of signal components received by the photodetector. b) Photograph of the PPG sensor in the nonworking (top) and working (bottom) conditions. c) Pulse signal tested by the PPG sensor in 5 s (left) and amplified single pulse signal diagram (right). d) PPG signals before and after exercise. e‐h) PPG signals under different illumination intensities of 8.16, 2, 0.423, and 0.055 mW cm^−2^, respectively.

The high performance and excellent stability of the perovskite photodetectors array offer it an opportunity for long‐term health monitoring applications. As shown in Figure S18a (Supporting Information), a pulse monitoring system was constructed, consisting of a PI film as the substrate, Au patterns as the electrodes, a green light‐emitting diode (LED) as the light source, the perovskite films array as the light‐sensing layer, and a thin parylene‐C film as the encapsulation layer. Figure S18b (Supporting Information) shows the photograph of the as‐prepared pulse monitoring system, and its corresponding circuit is displayed in Figure S18c (Supporting Information). The pulse monitoring system could be worn on the index finger and it works based on the photoplethysmography (PPG) technique, which records the pulse by analyzing luminous flux^[^ ^59^, ^60^ ^]^ (the inset of **Figure** **5**a). When it is working, the green light emitted from the system irradiates into the skin and is reflected by the vessels to the photodetector, and the light received by the photodetectors is very weak because of the absorption by the skin, bone, tissues, and blood. That requires the photodetectors to be sufficiently sensitive to weak light. As shown in Figure 5b, the pulse monitoring system is flexible and could form conformal contact with fingers, reducing the influence of light noise. When the LED was on, the current rose sharply and then remained stable with a small regular wave (PPG signal) (Figure S19, Supporting Information). Figure 5c shows the PPG signal measured in 5 s with the illumination intensity of 8.16 mW cm^−2^. The systolic peak and diastolic peak can be observed in individual pulses. As shown in Figure 5d, heart rate can also be monitored at rest and after severe exercise with a green illumination of 8.16 mW cm^−2^, and it is calculated to be 78 and 114 bpm, respectively. More importantly, the PPG signal can be recorded at an illumination intensity as low as 0.055 mW cm^−2^. As shown in Figure 5e–h, with decreasing the intensity from 8.16 to 0.055 mW cm^−2^, the PPG signal with systolic peak and diastolic peak can be obtained. This working light intensity of 0.055 mW cm^−2^ is low compared to previously reported work, as shown in Table S3 (Supporting Information). Furthermore, the PPG sensor consists of several independent photodetectors, which can simultaneously detect pulse signals. By simultaneously measuring and averaging the signals from multiple pixels in the array device, the accuracy of the pulse signal can be improved (Figure S20, Supporting Information).^[^ ^61^ ^]^ As shown in Figure S20a,b (Supporting Information), the signals from three pixels were simultaneously measured while attaching the device to a fingertip. Subsequently, the individual waveforms are averaged, which could effectively improve the quality of the pulse signal and eliminate the signals resulting from the shake or slide during the testing process (Figure S20c, Supporting Information). Similarly, the strategy can be applied for the improvement of the multiple pulse signals (Figure S20d, Supporting Information). Benefiting from the excellent stability of the perovskite photodetectors, the pulse monitoring system could be worn in daily life. Figure S21 (Supporting Information) exhibits the PPG signal measured by the systems that have been worn on the index finger for 12 h. The user wore the device on the index finger while carrying out ordinary tasks, including typewriting, wearing gloves, and using cutlery. For comparison, the reference device was worn for 12 h with greater care taken to shield it from impacts and friction. Both of them were able to measure the heart rate accurately, indicating their capability of long‐term pulse monitoring. Considering the bulk focusing optics required by the commercialized sensor, our flexible system with high sensitivity and simple structure exhibits great potential for low power consumption PPG sensors.

## 3 Conclusion

We demonstrated a novel one‐step vapor deposition method for all‐inorganic perovskite film arrays. Through regulating surface energy, growth temperature, and growth time, all‐inorganic perovskite films arrays can be deposited in various patterns with a selectivity of 96.7%. In addition, a perovskite film array with a resolution of 423 dpi was successfully deposited, which exhibited the potential application of the SEAPVD method in the integration of a high‐resolution optoelectronic device. The photoconductor‐typed photodetectors array demonstrated excellent photoelectric performance, showing a high on/off ratio of 13 887, a large responsivity of 47.5 A W^−1^, as well as outstanding chemical, environmental, and working stability. Moreover, it could be integrated into a PPG sensing system, realizing stable monitoring of pulse signals with an ultra‐low light intensity. These results expand the application of perovskite materials for long‐term wearable optoelectronic systems.

## 4 Experimental Section

### Preparation of the Patterned Substrate

First, the substrate was treated with the O_2_ plasma (PDC100B Plasma Cleaner) with 50 sccm O_2_ at 150 W for 180 s to get a hydrophilic surface. Second, the positive photoresist (S1813) was spun onto the substrate at 4000 rpm for 1 min and the patterned photoresist was obtained after exposure for 12 s and development (ZX238) for 30 s. And then a 20 nm SiO_2_ thin film was deposited on the substrate by magnetron sputtering (Kurt J. Lesker PVD75) at 200 W for 10 min. Subsequently, the substrate was placed into an octadecyl‐trichlorosilane (OTS) solution (OTS: n‐Hexane = 1:200) for 15 min. Finally, after removing from the OTS solution, the substrate was immediately put into the acetone for ultrasonic cleaning for 10 min and cleaned with Deionized (DI) water to remove the photoresist and residual solvent. To acquire the patterned substrate with different surface energies in the growth area, the as‐prepared substrate was immersed into the OTS solution with low concentration (OTS: n‐Hexane = 1:20 000) for 1–40 min. Finally, the substrate was taken out from the OTS solution and immediately put into acetone and DI water in turn.

### Vapor Deposition of Perovskite Films Array

The vapor deposition of the perovskite films array was carried out in the tube furnace (Thermcraft XST‐3‐0‐12‐1C) equipped with a vacuum pump (Edwards RV8) and adopts the “tube‐in‐tube” method conducive to maintaining the stability of flow, where the inner diameters of the outer tube and inner tube were 52 and 46 mm, respectively. The quartz boat filled with about 0.2 g CsPbBr_3_ powder was placed in the heating center of the tubular furnace and the patterned substrate was placed in the downstream area (14 cm away from the source). Next, the system was vacuumed, and then 40 sccm Ar was injected for 30 min to ensure the evacuation of the air. Subsequently, the system was heated from 30 to 430 °C in 30 min, held at 430 °C for 30 min, and naturally cooled down to room temperature. Finally, the perovskite film arrays were obtained. For the preparation of CsPbCl_x_Br_3‐x_ and CsPbBr_x_I_3‐x_, the evaporation source powder was obtained by mixing and fully grinding CsPbCl_3_ and CsPbBr_3_, CsPbBr_3,_ and CsPbI_3_ in different proportions, respectively, as shown in Figure S15a (Supporting Information).

### Integration of PPG Sensor

First, the interdigital electrodes were deposited on the 75 µm PI film by photolithography and magnetron deposition. To avoid the deformation of the PI film during lithography and the deposition of perovskite film, PI film was stuck on the glass. Second, patterned SiO_2_ was prepared by lithography and magnetron sputtering, whose windows were aligned with the as‐deposited interdigital electrodes. After the growth of perovskite film arrays, a commercial green LED was pasted next to the perovskite photodetector with silver paste and PI tape. Finally, the PPG sensor was encapsulated with 3 µm thick parylene‐C by a polymer organic vapor deposition system (MAGGIE MQP‐3001), which was first evaporated at 120 °C and then split at 680 °C and passed up to the vacuum chamber depositing on the samples.

### Characterization and Measurements

An optical microscope (Zeiss Observer Z1) and scanning electron microscopes (Hitachi SU8020 and Nova 450) were used to observe the micromorphology and EDS mappings of perovskite film arrays. X‐ray diffraction (X'Pert3 Powder) was employed for the phase analysis of perovskite. The fluorescence spectrum and lifetime were measured by Edinburgh FLS980. The absorption spectra of perovskite were obtained by a UV–Vis‐NIR spectrophotometer (Shimadzu UV 3600). LED was powered by a direct‐current power supply (Maynuo M8812). The photoelectric performance of the perovskite photodetector including I‐V and I‐T curves was acquired by an electrical test system consisting of an adjustable power supply (Stanford DS 345) and a current amplifier (Stanford SR 570).

## Conflict of Interest

The authors declare no conflict of interest.

## Supporting information

Supporting Information

## Data Availability

The data that support the findings of this study are available from the corresponding author upon reasonable request.
