# Supplementary material for: Surface Energy‐Assisted Patterning of Vapor Deposited All‐Inorganic Perovskite Arrays for Wearable Optoelectronics
Source: Adv Sci (Weinh). 2024 Apr 19;11(25):2402635. doi: 10.1002/advs.202402635 (PMC11220711; doi:10.1002/advs.202402635)
Supplement: Supplementary file 1 — Supporting Information [file ADVS-11-2402635-s001.pdf]

ADVANCED SCIENCE
Open Access

Supporting Information

for *Adv. Sci.*, DOI 10.1002/advs.202402635

Surface Energy-Assisted Patterning of Vapor Deposited All-Inorganic Perovskite Arrays for Wearable Optoelectronics

Zhangsheng Xu, Xiaojun Pan, Hui Lu, Qiuchun Lu, Yegang Liang, Zeping He, Yizhi Zhu, Yang Yu, Wenqiang Wu, Xun Han* and Caofeng Pan**

Supporting Information

Surface energy-assisted patterning of vapor deposited all-inorganic perovskite arrays for wearable optoelectronics

Zhangsheng Xu, Xiaojun Pan, Hui Lu, Qiuchun Lu, Yegang Liang, Zeping He, Yizhi Zhu, Yang Yu, Wenqiang Wu, Xun Han*, Caofeng Pan**

Note S1: Calculation of surface free energy.

In Young's equation, the interfacial energy between solid and liquid (γ_{sl}) can be expressed by [1]:

$$\gamma_{sl} = \gamma_s - \gamma_l \cos\theta \qquad (1)$$

where θ is the contact angle, γ_s and γ_l is the surface free energy of solid and liquid, respectively. In addition, according to the Owens-Wendt-Rabel and Kaelble (OWRK) method, surface free energy (γ) can be assumed to consist of the dispersive component (γ^d) and the polarity component (γ^p) and γ_{sl} can be expressed as the formula [2]:

$$\gamma_{sl} = \gamma_s + \gamma_l - 2(\gamma_s^d \gamma_l^d)^{1/2} - 2(\gamma_s^p \gamma_l^p)^{1/2} \qquad (2)$$

where γ_s^d and γ_s^p represent the dispersive and the polarity component of solid; besides, γ_l^d and γ_l^p are the dispersive and the polarity component of liquid. Combining the above two equations, we can obtain the equations:

$$\gamma_l (1+\cos\theta) = 2(\gamma_s^d \gamma_l^d)^{1/2} + 2(\gamma_s^p \gamma_l^p)^{1/2} \qquad (3)$$

According to equation 3, the dependence of $\gamma_l (1+\cos\theta)/2(\gamma_l^d)^{1/2}$ on $(\gamma_l^p/\gamma_l^d)^{1/2}$ is drawn to obtain γ_s^d and γ_s^p from the slope and the intercept of the fitting line. γ_{sl} can be acquired by ($\gamma_s^d + \gamma_s^p$). The solvents used in the calculation of surface free energy are displayed in Table S1 [3].

Note S2: The process of the patterned substrate with different surface energies

The fabrication of the patterned substrate with different surface energies in the growth area as below:

1. Preparation of the patterned substrate: Firstly, the substrate was treated by the O_2 plasma (PDC100B Plasma Cleaner) with 50 sccm O_2 at 150 W for 180 s to get a hydrophilic surface. Secondly, the positive photoresist (S1813) was spun onto the substrate at 4000 rpm for 1 min and the patterned photoresist was obtained after exposure for 12 s and development (ZX238) for 30 s. And then SiO_2 thin film was deposited on the substrate by magnetron sputtering (Kurt J. Lesker PVD75) at 200 W for 10 min. Subsequently, the substrate was placed into an octadecyl-trichlorosilane (OTS) solution (OTS: n-Hexane =1:200) for 15 min. Finally, after removing from the OTS solution, the substrate was immediately put into the acetone for ultrasonic cleaning for 10 min and cleaned with Deionized (DI) water to remove the photoresist and residual solvent.
2. Modulation of the surface energy in the growth area: Due to that the reaction between hydroxyl on the substrate and OTS is not instantaneous (Figure S4), premise modulation of surface energy can be achieved through controlling the self-assembly time of OTS treatment. The as-prepared substrate was immersed into the OTS solution with low concentration (OTS: n-Hexane =1:20000) for 1-40 min to obtain different surface energies. Due to the complete reaction in the non-growth area, the OTS only reacted with the hydroxyl group in the growth area, and low concentration OTS solution simultaneously alleviated the excessive reaction between OTS and the hydroxyl group. Finally, the substrate was cleaned with acetone and DI water immediately after removal from OTS.

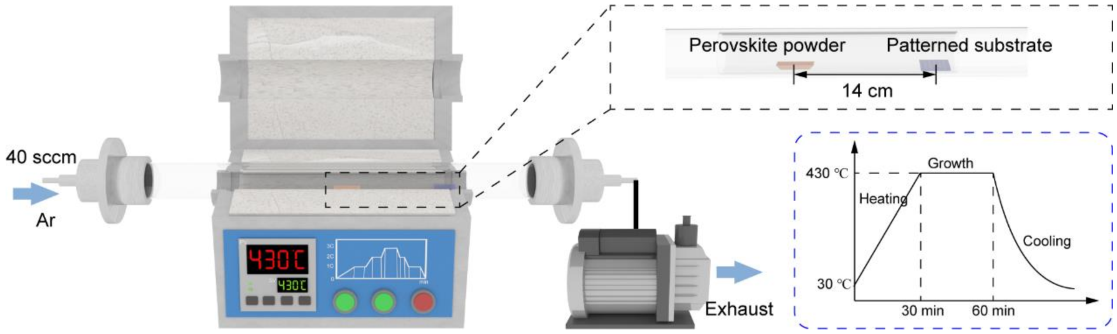

Figure S1. Schematic illustration of the perovskite film deposition in the Tube furnace. The inset is a magnified schematic illustration of the placement position of perovskite powder and patterned substrate (black box) and temperature versus time curve of the tubular furnace (blue box).

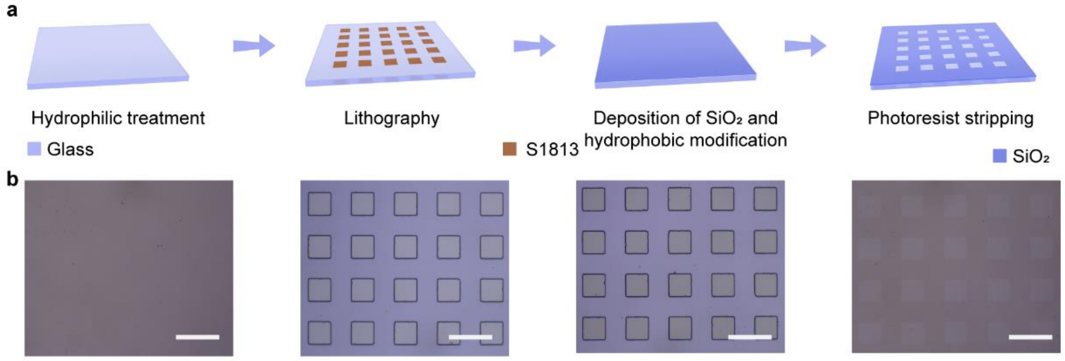

Figure S2. Preparation of the patterned substrate. a) Schematic diagram about the preparation of the patterned substrate. b) Optical images of the substrate corresponding to (a). The scale bar is 200 μm.

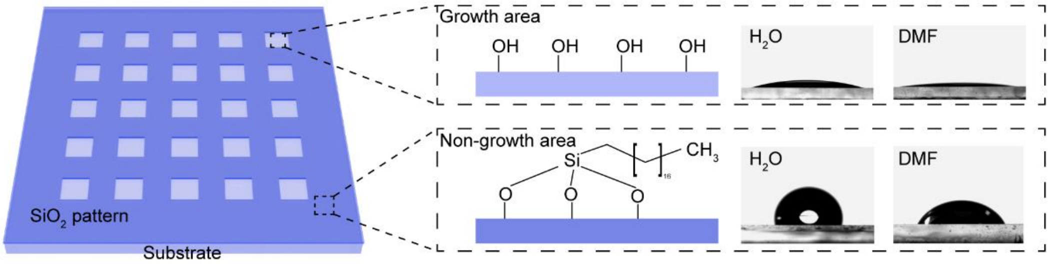

Figure S3. The contact angle of H_2O and DMF in the growth and non-growth areas, respectively.

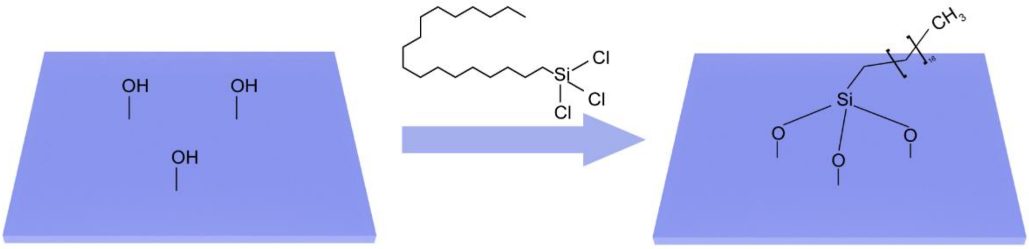

Figure S4. Schematic illustration of the reaction between OTS and hydroxyl groups on the substrate surface.

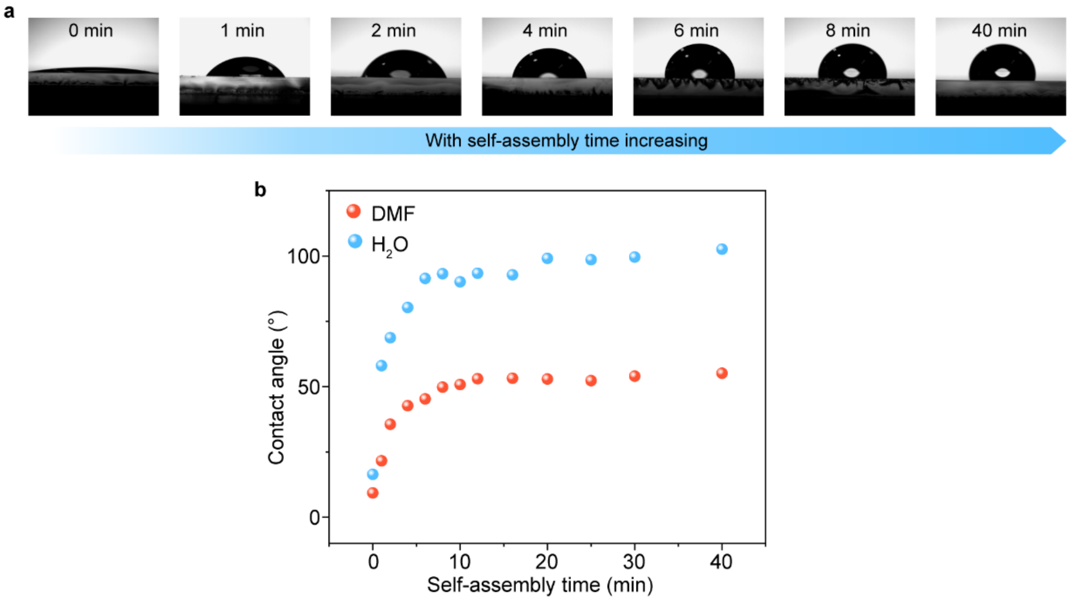

Figure S5. a) The contact angle of H_2O with self-assembly time increasing. b) The dependence of the contact angle of H_2O (red) and DMF (red) on self-assembly time.

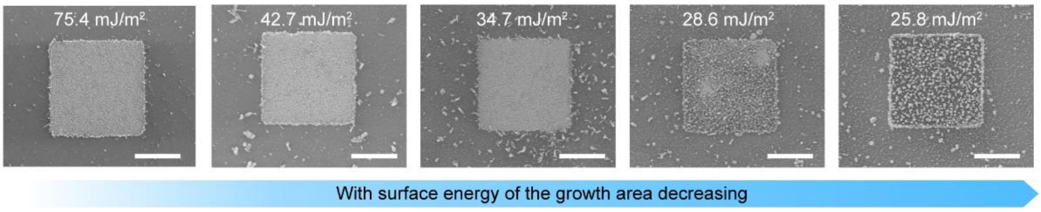

Figure S6. SEM images of the patterned perovskite film with the surface energy growth area of 75.4, 42.7, 34.7, 28.6, and 25.8 mJ/m^2, respectively. The scale bar is 50 μm.

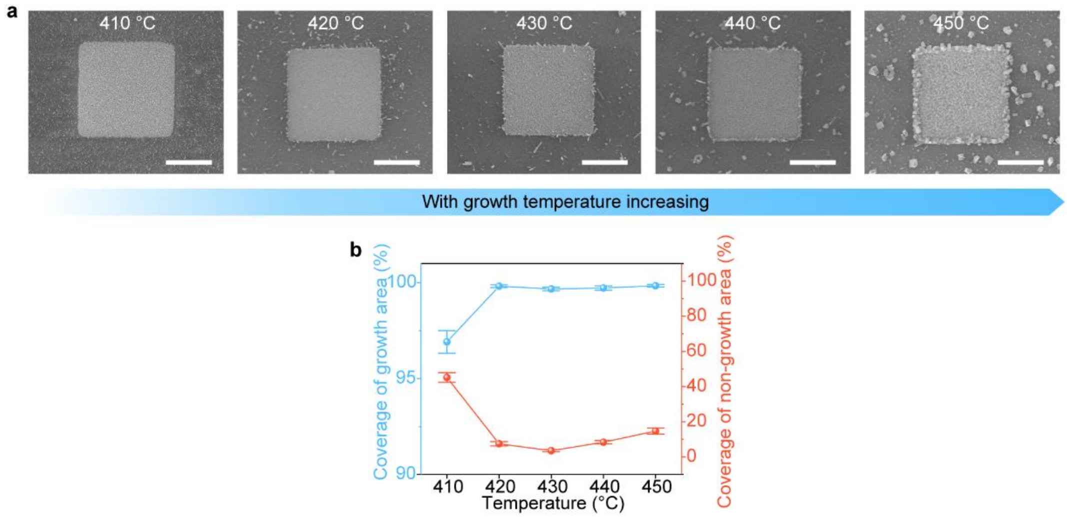

Figure S7. a) SEM images of the patterned perovskite film fabricated at 410 ℃ to 450 ℃. The scale bar is 50 μm. b) The dependence of coverage of the growth area (blue) and the non-growth area (red) on the growth temperature.

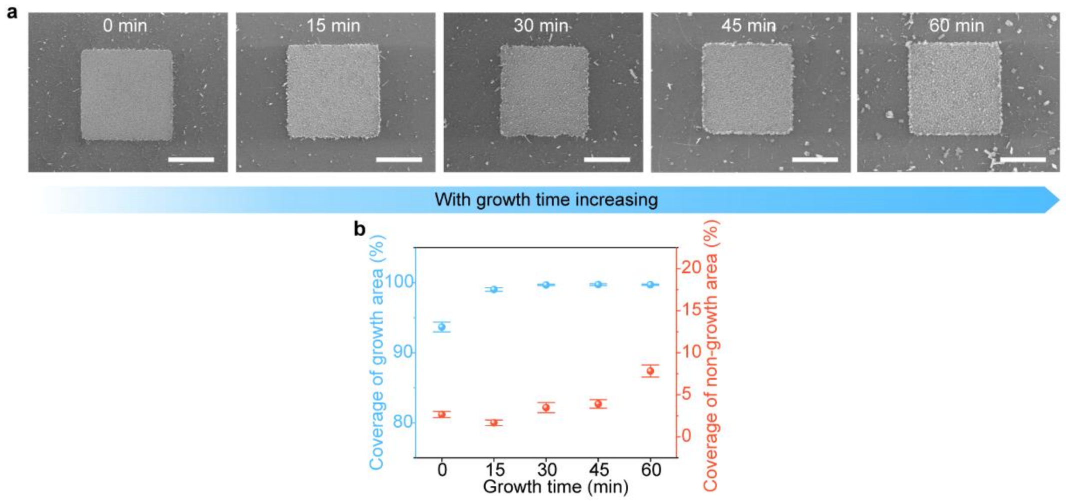

Figure S8. a) SEM images of the patterned perovskite film fabricated at 430 ℃ for 0, 15, 30, 45, and 60 min, respectively. The scale bar is 50 μm. b) The dependence of coverage of the growth area (blue) and the non-growth area (red) on the growth time.

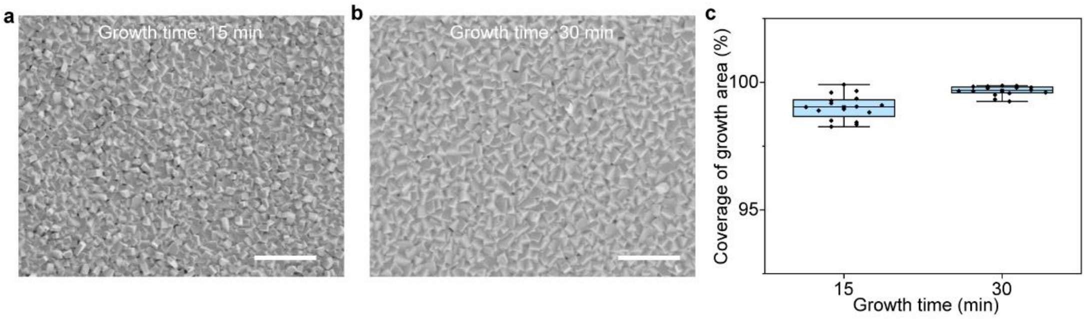

Figure S9. SEM images of perovskite films prepared at 430 °C for a) 15 min and b) 30 min. The scale bar is 10 μm. c) The coverage of the growth area with growth time of 15 min and 30 min.

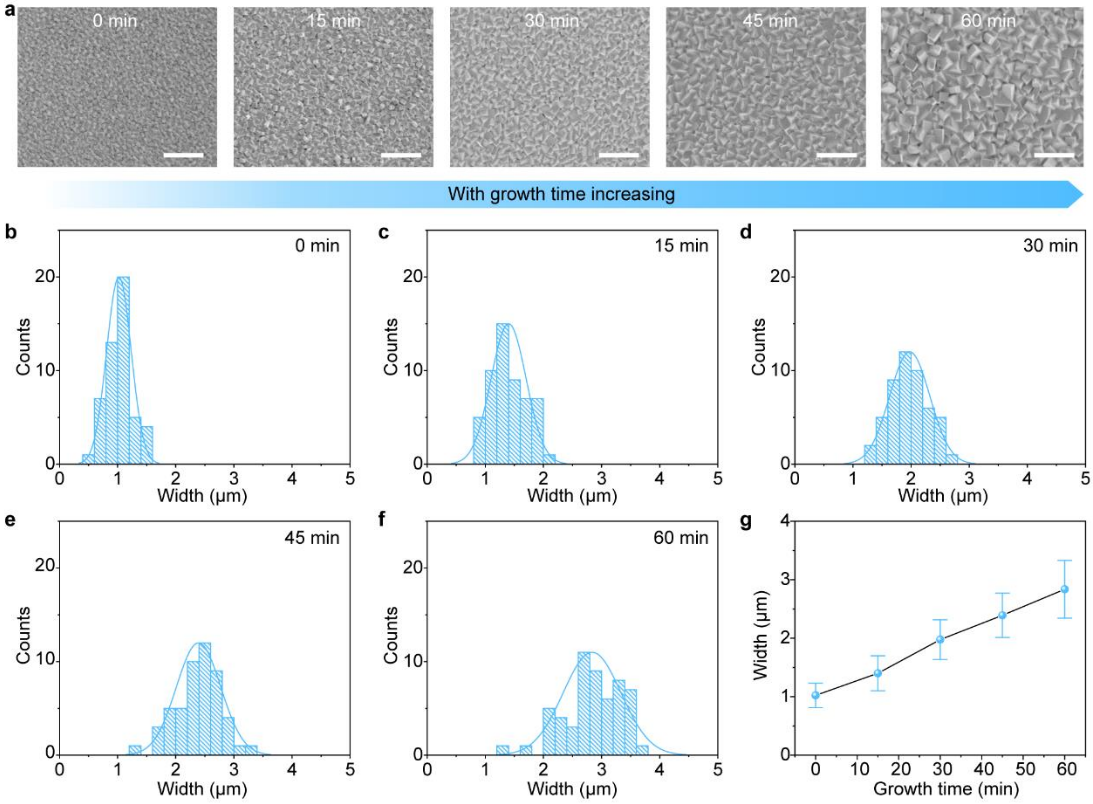

Figure S10. a) Magnified SEM images of the perovskite film on the growth area with the growth time of 0, 15, 30, 45, and 60 min, respectively. The scale bar is 10 μm. b-f) The width distribution of perovskite particles with the soaking time of 0, 15, 30, 45, and 60 min, respectively. g) The dependence of the perovskite particle width on the growth time.

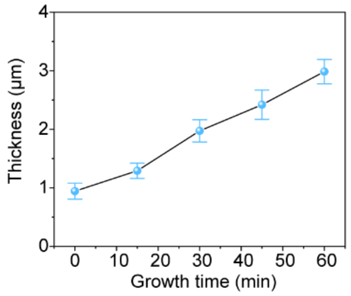

Figure S11. a) The dependence of the thickness of perovskite film on the growth time.

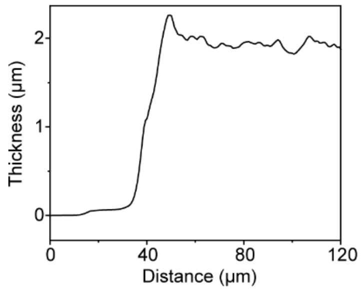

Figure S12. The thickness of $CsPbBr_3$ film,

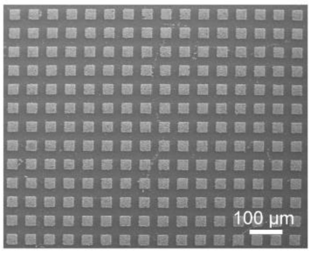

Figure S13. SEM image of $CsPbBr_3$ films array with an interval of 60 μm.

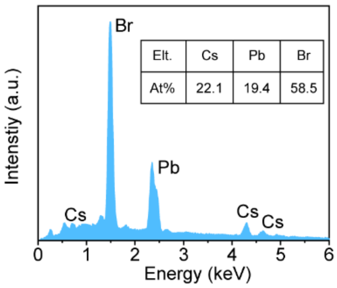

Elt.	Cs	Pb	Br
At%	22.1	19.4	58.5

Figure S14. EDS analysis of $CsPbBr_3$ film.

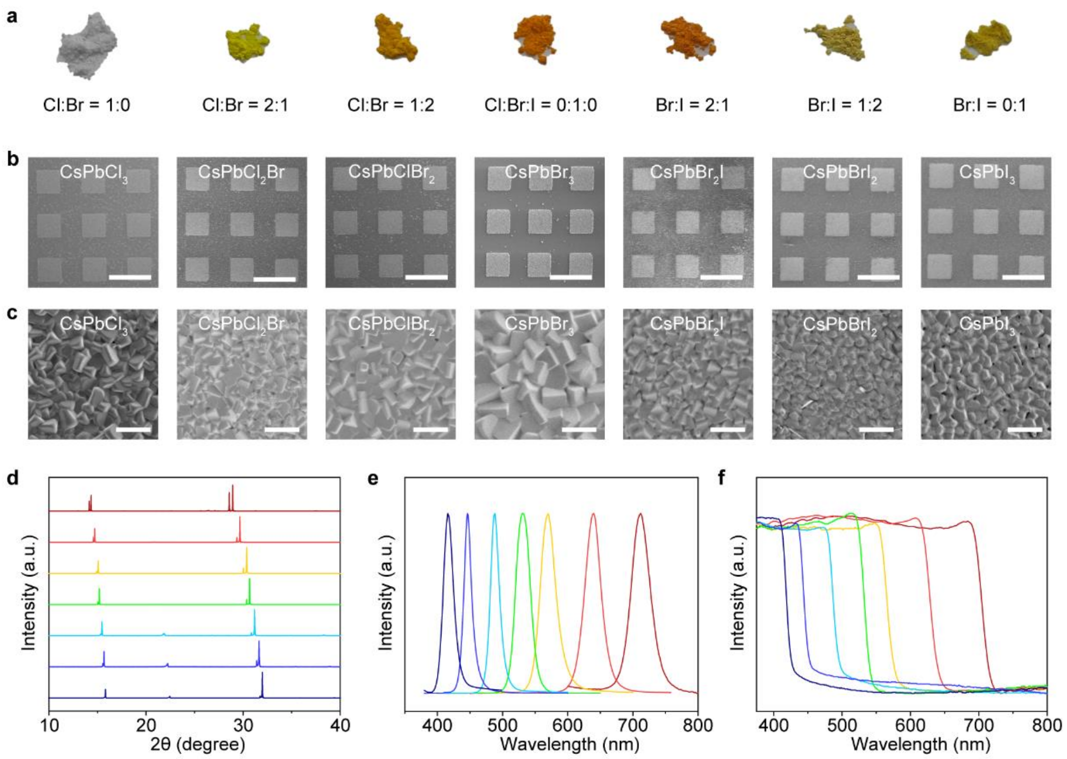

Figure S15. a) $CsPbX_3$ powder with different halogen ratios. b) SEM images of $CsPbX_3$ film arrays. The scale bar is 200 µm. c) Partially magnified SEM images of $CsPbX_3$ films. The scale bar is 5 µm. d) XRD, e) PL spectrum and absorption spectrum of the as-fabricated $CsPbX_3$ with different halogen ratios.

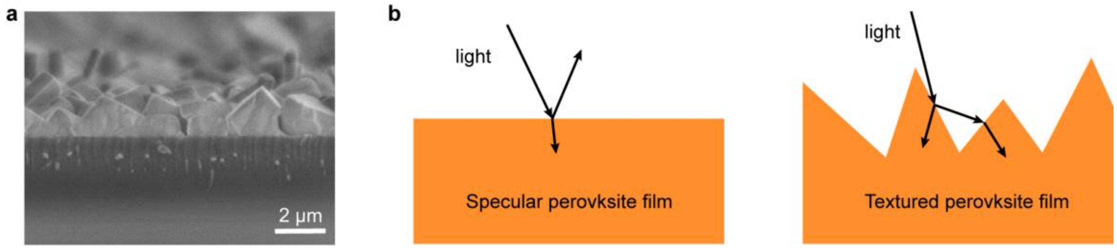

Figure S16. (a) SEM cross-sectional view image of the perovskite film. (b) Schematic illustration of light reflection behavior on the specular and textured surface.

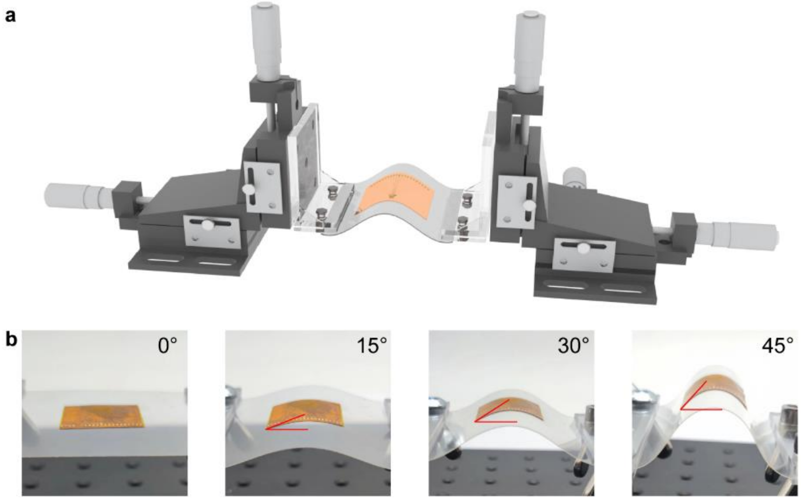

Figure S17. a) Schematic diagram of the device bending. b) Photograph of the device bending at 0°, 15°, 30°, and 45°.

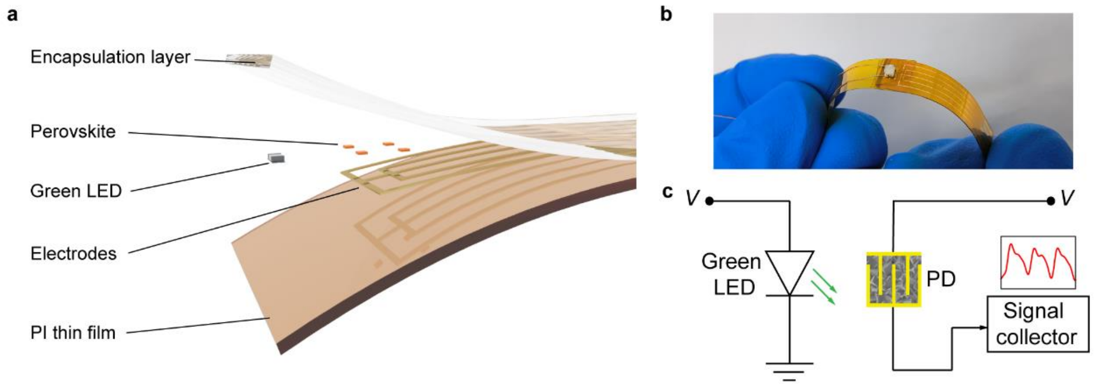

Figure S18. a) Schematic diagram of the perovskite PPG sensor structure. b) Photograph of the bending device. c) Circuit of the PPG sensor.

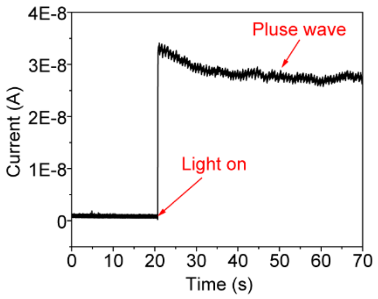

Figure S19. Current-time trace of the PPG sensor.

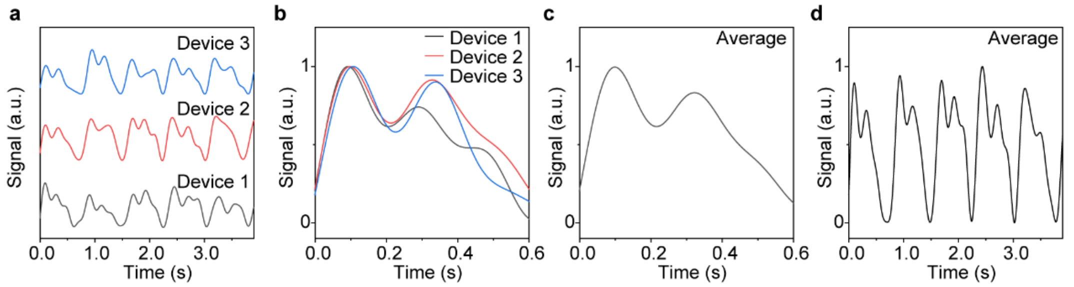

Figure S20. (a) PPG signals measured simultaneously by three devices. (b) Single pulse signal measured by Device 1-3. (c) Averaged signals for Device 1-3. (d) Average signal of multiple pulse waves.

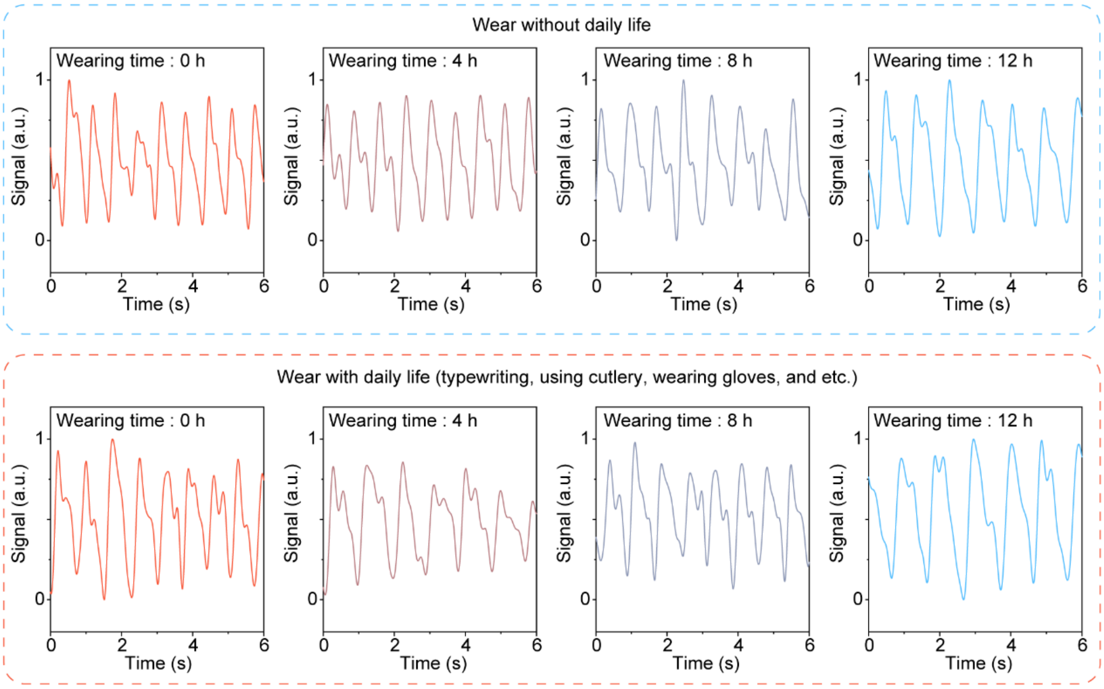

Figure S21. PPG signals measured after wearing for 12 h with and without daily life.

Table S1. The surface free energy and its dispersive and polarity component of H_2O and DMF

Liquid	γ_l^p (mJ/m^2)	γ_l^d (mJ/m^2)	γ_l (mJ/m^2)
H_2O	43.7	29.1	72.8
DMF	4.88	32.42	37.3

Table S2. The comparison of photoelectronic performance between the all-inorganic photodetector in this work and others reported previously.

**Material**	**Method**	**On/off ratio**	**R (A/W)**	**D* ($\times10^{12}$ Jones)**	**Array**	**Response/ decay time (ms)**	**Effective areas**	**Dark current density (A/cm^2)**	**Environmental stability**	**Mechanical stability**	**Working stability**	**Ref.**
$CsPbBr_3$ film	Vapor-deposition	13887	11.56 (1V)/47.50 (3V)	38.3 (1V)/62.4 (3V)	Yes	0.81/2.03	0.0001 cm^2/pixel	3.25×10^{-5} (3 V)/ 3.93×10^{-6} (1 V)	More than a month	10000 cycles	10 h	This work
$CsPbBr_3$ film	Solution-phase	483	2.1 (9V)	-	Yes	0.25/0.45	0.01 cm^2	-	3 month	2000 cycles	-	4
$CsPbBr_3$ single crystal	Low-temperature solution	100000	0.028	0.17	No	230/60	0.06 cm^2	~ 1.67×10^{-7} (6 V)	-	-	50 min	5
$CsPbBr_3$ nanosheets	Spin-coating	2300	0.0449 (0.2V)	0.00064	Yes	48/18	0.04 cm^2	~ 9.6×10^{-6} (10 V)	-	1500 cycles	-	6
$CsPb(Br/I)_3$ nanorods	Solution-phase	2000	-	-	No	0.68/0.66	> 0.0002 cm^2	-	-	-	-	7
$CsPbBr_3$ film	Vacuum-assisted drop-casting patterning process	~	3.15 (5V)	3.94	Yes	8/6.5	0.0001 cm^2/pixel	~ 3.3×10^{-4} (5 V)	6 weeks	5000 cycles	25 min	8
$CsPbI_3$ nanowires	One-step solution process		0.745	0.0346	No	-	~ 2.5×10^{-8} cm^2	-	-	-	-	9
$CsPbCl_3$ films	Antisolvent-induced crystallization		2.11	5.6	No	77/63	0.16 cm^2	~ 1.25×10^{-7} (5.65 V)	5 month	-	35 min	10
$CsPbBr_3$ microwires	Two-step method of facile phase induced growth	5669	5.18	2.74	Yes	275/550	1.44×10^{-5} cm^2	~ 6.63×10^{-4} (5 V)	-	-	60% after 30 h	11
$CsPbBr_3$ film	Vapor-deposition	380	0.375	0.296	No	0.28/0.67	4×10^{-5} cm^2	~ 3.3×10^{-4} (5 V)	60 days	1000 cycles		12
$CsPbBr_3$ film	Freeze-drying	200	0.05	0.4	Yes	0.4/2.3	0.002 cm^2	~5×10^{-4} (3 V)	-	-	-	13

Table S3. The comparison of photoelectronic performance between PPG sensors in this work and others reported previously.

Materials	Structure	Working area (cm^2)	Working principle	Flexibility	Working light intensity	Ref
$Cs_{0.07}FA_{0.93}PbI_3$	Photovoltaic transistor	>0.01 cm^2	Reflection mode	No	1 mW	14
0ZnO/PTzNTz-BOBO:$PC_{70}BM$	Photodiode	0.04 cm^2	Reflection mode	Yes	-	15
Graphene	Photoconductor	0.01 cm^2	Reflection/transmission mode	Yes	0.01 mW/cm^2 (Indoor light)	16
PTB7/$PC_{71}BM$	Photodiode	0.21 cm^2	Transmission mode	No	20.1 mW/cm^2 (green), 5.83 mW/cm^2(red)	17
**$CsPbBr_3$**	**Photoconductor**	**0.0001 cm^2**	**Reflection mode**	**Yes**	**0.055 mW/cm^2**	**Our work**

References

[1] N. K. Adam, *Nature* **1957**, 180, 809-810.

[2] M. Kalin, M. Polajnar, *Appl. Surf. Sci.* **2014**, 293, 97-108.

[3] Y.-q. Zhu, C.-x. Yu, Y. Li, Q.-q. Zhu, L. Zhou, C. Cao, T.-t. Yu, F.-p. Du, *Pest Management Science* **2014**, 70, 462-469.

[4] F. Cao, D. Yu, X. Li, Y. Zhu, Z. Sun, Y. Shen, Y. Wu, Y. Wei, H. Zeng, *J. Mater. Chem. C* **2017**, 5, 7441-7445.

[5] M. I. Saidaminov, M. A. Haque, J. Almutlaq, S. Sarmah, X.-H. Miao, R. Begum, A. A. Zhumekenov, I. Dursun, N. Cho, B. Murali, O. F. Mohammed, T. Wu, O. M. Bakr, *Adv. Opt. Mater.* **2017**, 5, 1600704.

[6] W. Deng, H. Huang, H. Jin, W. Li, X. Chu, D. Xiong, W. Yan, F. Chun, M. Xie, C. Luo, L. Jin, C. Liu, H. Zhang, W. Deng, W. Yang, *Adv. Opt. Mater.* **2019**, 7, 1801521.

[7] X. Tang, Z. Zu, H. Shao, W. Hu, M. Zhou, M. Deng, W. Chen, Z. Zang, T. Zhu, J. Xue, *Nanoscale* **2016**, 8, 15158-15161.

[8] W. Wu, X. Han, J. Li, X. Wang, Y. Zhang, Z. Huo, Q. Chen, X. Sun, Z. Xu, Y. Tan, C. Pan, A. Pan, *Adv. Mater.* **2021**, 33, 2006006.

[9] Y. Chen, G. Chen, Z. Zhou, X. Li, P. Ma, L. Li, W. Yin, H. Zeng, G. Zou, *Adv. Funct. Mater.* **2021**, 31, 2101966.

[10] Z. Zhu, W. Deng, W. Li, F. Chun, C. Luo, M. Xie, B. Pu, N. Lin, B. Gao, W. Yang, *Adv. Mater. Interfaces* **2021**, 8, 2001812.

[11] G. Tong, M. Jiang, D. Y. Son, L. K. Ono, Y. Qi, *Adv. Funct. Mater.* **2020**, 30, 2002526.

[12] G. Tong, H. Li, D. Li, Z. Zhu, E. Xu, G. Li, L. Yu, J. Xu, Y. Jiang, *Small* **2018**, 14, 1702523.

[13] J. Xue, Z. Zhu, X. Xu, Y. Gu, S. Wang, L. Xu, Y. Zou, J. Song, H. Zeng, Q. Chen, *Nano Lett.* **2018**, 18, 7628-7634.

[14] Y. Li, G. Chen, S. Zhao, C. Liu, N. Zhao, *Sci. Adv.* **2022**, 8, eabq0187.

[15] H. Jinno, T. Yokota, M. Koizumi, W. Yukita, M. Saito, I. Osaka, K. Fukuda, T. Someya, *Nat. Commun.* **2021**, 12, 2234.

[16] E. O. Polat, G. Mercier, I. Nikitskiy, E. Puma, T. Galan, S. Gupta, M. Montagut, J. J. Piqueras, M. Bouwens, T. Durduran, G. Konstantatos, S. Goossens, F. Koppens, *Sci. Adv.*, 5, eaaw7846.
[17] C. M. Lochner, Y. Khan, A. Pierre, A. C. Arias, *Nat. Commun.* **2014**, 5, 5745.
